# Polyphenols Targeting NF-κB Pathway in Neurological Disorders: What We Know So Far?

**DOI:** 10.7150/ijbs.90982

**Published:** 2024-01-27

**Authors:** Abdullah Al Mamun, Chuxiao Shao, Peiwu Geng, Shuanghua Wang, Jian Xiao

**Affiliations:** 1 Central Laboratory of The Sixth Affiliated Hospital of Wenzhou Medical University, Lishui People's Hospital, Lishui City, Zhejiang 323000, China.; 2 Molecular Pharmacology Research Center, School of Pharmaceutical Sciences, Wenzhou Medical University, Wenzhou 325000, China.; 3 Department of Wound Healing, The First Affiliated Hospital of Wenzhou Medical University, Wenzhou 325000, China.

**Keywords:** Polyphenols, NF-κB pathway, neurological disorders, Neuroinflammation, Alzheimer's disease, Parkinson's disease, spinal cord injury

## Abstract

Polyphenolic compounds have shown promising neuroprotective properties, making them a valuable resource for identifying prospective drug candidates to treat several neurological disorders (NDs). Numerous studies have reported that polyphenols can disrupt the nuclear factor kappa B(NF-κB) pathway by inhibiting the phosphorylation or ubiquitination of signaling molecules, which further prevents the degradation of IκB. Additionally, they prevent NF-κB translocation to the nucleus and pro-inflammatory cytokine production. Polyphenols such as curcumin, resveratrol, and pterostilbene had significant inhibitory effects on NF-κB, making them promising candidates for treating NDs. Recent experimental findings suggest that polyphenols possess a wide range of pharmacological properties. Notably, much attention has been directed towards their potential therapeutic effects in NDs such as Alzheimer's disease (AD), Parkinson's disease (PD), cerebral ischemia, anxiety, depression, autism, and spinal cord injury (SCI). Much preclinical data supporting the neurotherapeutic benefits of polyphenols has been developed. Nevertheless, this study has described the significance of polyphenols as potential neurotherapeutic agents, specifically emphasizing their impact on the NF-κB pathway. This article offers a comprehensive analysis of the involvement of polyphenols in NDs, including both preclinical and clinical perspectives.

## Introduction

A neurological disorder (ND) is any pathological condition affecting the nervous system. Various symptoms may arise from biochemical, structural, or electrical disorders in the brain, spinal cord, or peripheral nerves 1. The increasing prevalence of NDs due to population aging poses substantial challenges to the long-term sustainability of healthcare systems, especially in low- and middle-income nations 2. As global populations age, changes in brain structure, biochemistry, and peripheral nerves may cause various symptoms, including poor coordination, muscle weakness, pain, paralysis, loss of feeling, convulsions, disorientation, and changes in consciousness, which can be attributed to the aging population 3. Non-communicable NDs, such as multiple sclerosis, Alzheimer's disease (AD), Parkinson's disease (PD), migraines, non-migraine headaches, and epilepsy, are causing global public health concerns about mortality and cognitive deficiencies. The increasing prevalence of age-related disorders among older adults has led to significant social and economic implications, highlighting the need for effective treatment options. This concern is expected to escalate in the coming years 4-8.

The nuclear factor kappa-B (NF-κB) pathway regulates immunological responses, cell division, and apoptosis. Neurons and surrounding cells also utilize it for various purposes, including nervous system formation, cellular response coordination, and brain-specific processes such as synaptic signaling. *In vivo*, research experimentation suggests that modulation of the NF-κB pathway could assist in treating conditions such as ischemic stroke, physical brain damage, and NDs such as AD and PD. These functions are evident in the nervous system and surrounding cells 9. NF-κB, an important player in neural circuit structure and function, is regulated by various signaling pathways. Its activity is influenced by neurotransmitters, neurotrophic factors, cytokines, electrical activity, and oxidative stress (OS). The components of NF-κB can influence cognitive processes and behavioral outcomes through target gene regulation, which can potentially mitigate neuronal cell death by stimulating the synthesis of antiapoptotic peptides. NF-κB is also associated in many disease pathogenesis, including epilepsy, stroke, AD, and PD 10. Genetic research indicates that the continuous activation of NF-κB is crucial for neuron survival, while pharmacological inhibition of the IKK complex results in neuronal apoptosis. NF-κB pathway activation is observed to protect neurons against the toxicity caused by Aβ, thereby potentially disrupting neuroprotective mechanisms in AD patients. Hyperactivation of NF-κB is correlated to NDs including ischemia, PD, and p53 transcription-mediated neuronal death 11.

Research has been conducted to understand the molecular mechanisms and treatment targets of neurodegenerative disorders. The optimal effectiveness of neuroprotection strategies is achieved when they effectively engage with the pathophysiological transition process, mitigating or delaying neurodegeneration. Natural substances have historically been known for their therapeutic advantages. The study of bioactive polyphenols from natural sources is gaining attention due to their potential health and medicinal benefits 12-15. Research studies have shown that polyphenols possess neuroprotective properties, preserving neurons from neurotoxicity, suppressing inflammation, and improving memory, learning, and cognitive function. Polyphenols, a diverse group of natural antioxidants, have a complex chemical tapestry with various biological effects 16. Multiple hydroxyl groups on aromatic rings and exhibit sub-groups such as flavonoids, tannins, and lignans characterize them. Each subgroup's chemical fingerprint influences biological interactions 17. Pharmaceutical research often employs an in-depth approach to acquiring polyphenols, considering many criteria, such as their natural occurrence, degree of purity, special requirements, and sustainability 18. Fruits, vegetables, cereals, and drinks are natural sources that include various polyphenols. However, the amount and quality of these polyphenols might vary 19. Studies investigating certain compounds tend to focus on particular polyphenols, and standardized extracts guarantee constant composition 20. Cost and sustainability are crucial factors, which may be achieved by extensive culture, microbial production, and adherence to quality standards to maintain consistency. Chemical synthesis provides an alternate method but encounters difficulties such as intricacy, bioavailability, and environmental consequences. The practicality and sustainability of generating particular polyphenols are enhanced by advancements in organic synthesis and biocatalysis 21.

Polyphenols exert promising antioxidant properties, protecting cells against oxidative damage and chronic ailments 22. In addition, they possess anti-inflammatory properties via regulating inflammatory pathways and enhance cardiovascular well-being by improving blood vessel functionality and reducing LDL cholesterol levels 23. Additionally, they possess neuroprotective properties, which may aid in treating neurodegenerative disorders 12,24. Recent research suggests polyphenols can decrease OS and inflammation, progress protective signaling, and activate genes that produce antioxidant enzymes and neurotrophic factors. These pathways, mainly NF-κB and Nrf-2/ARE pathways, maintain cerebral homeostasis and confer a vital role in adaptation to neural stress, thus preventing the development of NDs 25,26.

To ensure the inclusion of the most appropriate articles in this review, an in-depth search was carried out on prominent medical, biological, and chemical databases, including Scopus, PubMed, and Web of Science. The search used the keywords listed below: "Polyphenols, NF-κB pathway, neurological disorders, and neuroinflammation." Furthermore, the secondary keywords used were "Alzheimer's disease, Parkinson's disease, and Spinal cord injury." This review explores the potential of polyphenols in treating various diseases by modulating NF-κB pathways and their impact on NDs. It aims to understand the mechanisms behind polyphenols' effects, specifically their effect on the NF-κB pathway. The review also aims to evaluate the neuroprotective properties of polyphenols in various NDs and to assess their therapeutic capacity and potential use as a remedy.

## NF-κB biology associated with NDs

NF-κB can be activated through the classical/canonical and the alternative/non-canonical NF-κB pathway. The canonical pathway is widely researched and can be initiated by stimuli such as lipopolysaccharide (LPS), antigens, IL-1 receptors, TLRs, and TNF receptors. This response is triggered by pro-inflammatory mediators and the activation of the T-cell or B-cell receptor 27,28. The IKK complex, comprising IKKα, IKKβ, and NEMO, inhibits NF-κB by phosphorylating IκB proteins, releasing cytoplasmic NF-κB and allowing its translocation into the nucleus for transcriptional activity. The IKK complex serves as a convergence point for the canonical pathway, while the noncanonical route has specific ligands like CD40L and BAFF. NF-κB-inducing kinase (NIK) selectively phosphorylates IKKα, processing p100 into p52 and subsequently translocating to the nucleus through dimerization with RelB, initiating the activation of noncanonical NF-κB target genes 29,30.

NF-κB transcription factors are essential in the central nervous system (CNS) for physiological processes, including neurogenesis, neurogenesis, and synaptic plasticity, which are linked to memory and learning 31,32. They also work as a protective mechanism in contrast to several forms of injury, such as excitotoxicity, oxidative stress (OS), and amyloid β peptide toxicity. NF-κB also functions as a cellular defense program, aiding in the recovery of apoptotic cortical neurons. NF-κB is constitutive and has a dual function in neuronal injury and neuroprotection processes within neuron cell bodies 33. However, it is imperative to note that NF-κB is inactive at the synapse. The transportation of NF-κB to the nucleus of a neuron cell only occurs upon its activation 27,34.

The expression of NF-κB is found across various brain regions, indicating its widespread presence. NF-κB activation in neurons is consistently present and linked to processing neuronal information. The NF-κB pathway can regulate the process of gene transcription, specifically for genes involved in various cellular functions, such as cytokines, adhesion molecules, chemokines, proinflammatory enzymes, transcription factors, and other related factors. This regulatory mechanism allows NF-κB to influence neuronal survival 35. In the context of neuronal insult, the continuous activation of NF-κB inside neuronal cell bodies has been shown to have neuroprotective effects against many forms of injury and to modulate inflammatory responses within the neuronal system. In addition to neurons, glial cells and cerebral blood vessels have a significant presence of NF-κB transcription factors. The multifaceted roles of NF-κB also include regulating inflammatory responses within the neuronal milieu. NF-κB transcription factors are prevalent inside the brain, showcasing various activities. A correlation exists between the activation of NF-κB pathway by inflammatory mediators and the development of several disorders inside the CNS. The expression of RelA and c-Rel has distinct impacts on neuronal survival. The expression of c-Rel in the CNS plays a pivotal role in inhibiting programmed cell death (apoptosis) and mitigates age-related behavioral changes. Furthermore, the many subunits that create NF-κB dimers can regulate neuroinflammation, provide neuronal protection, and induce neurotoxicity 27.

Research indicates that NF-κB dysregulation is linked to neurodegenerative processes after trauma or ischemia and in individuals with neurodegenerative disorders 36. Neuroinflammatory chemicals significantly influence the pathogenesis of central nervous system disorders. The differential activation of NF-κB dimers determines the response of neurons to external stimuli. The RelA subunit, a component of NF-κB, initiates neurodegenerative processes from ischemia insults, Aβ toxicity, or glutamate 37-39. Therefore, these findings suggest that NF-κB has potential as a therapeutic target for mitigating neurodegeneration in the dopaminergic system. However, additional research is required to ascertain the most appropriate strategy and medication for treating NDs 40.

## Bioavailability of polyphenols

Bioavailability refers to the rate and duration at which the active compound or active moiety is absorbed from a drug substance and becomes accessible at the site of application. Assessment of the bioavailability of polyphenolic compounds is based not only on their transmembrane potential but also on their structural configuration. Most dietary polyphenolic compounds are metabolized in the gut and further methylated and transformed into sulfation and glucuronide metabolites by the liver enzymes 41. Regrettably, several researchers are still searching for food-producing flavonoids that influence the biological role of human health, and inappropriate compounds are likely to be studied. Polyphenols are less durable in cell culture medium than in organic solvents or water 42, suggesting that they are highly degradable in the natural environment, leading to poor bioavailability and a considerable reduction in biological activity. In the case of tea polyphenols, such as (-)-epicatechin, (-)-epicatechin gallate (ECG), (-)-epigallocatechin (EGC), and (-)-epigallocatechin gallate (EGCG), low lipid solubility contributes to their poor transmembrane capacity 43 and intestinal instability, rendering them strongly vulnerable to OS 44.

Furthermore, the bioavailability of polyphenols depends on multi-drug resistance proteins on the cell membrane surface 45 and p-glycoproteins 46. Recent studies involving human models considered the evidence on absorption, distribution, metabolism, and bioavailability. They regarded maximum plasma concentration (C_max_), plasma concentration-time curve (PCTC), time to reach C_max_, relative urinary excretion (RUE), and elimination half-life (EHL) as significant polyphenol indices. Gallic acid has been shown to possess the highest absorption capacity of all polyphenols 47.

### Phenolic acids

Phenolic acids can be categorized into two large groups: cinnamic acid and benzoic acid derivatives. An extensive range of fruits and vegetables has been reported to contain phenolic acids in various amounts; e.g., hydroxycinnamic acid concentration has been determined to be greater than hydroxybenzoic acid concentration. Compared to flavonoids or benzoic acid derivatives, hydroxycinnamic acids contribute more to the overall consumption of polyphenols. The increased consumption of this phenolic acid is attributed to coffee, which has a very high concentration of hydroxycinnamic acid. Many laboratory and epidemiological studies have reported the protective action of phenolic acids against different degenerative diseases 48,49. For instance, Kaliora et al. mentioned the bioavailability and pharmacokinetics of gallic acid relative in healthy humans. In this study, 125 mL of black Assam tea was made up to 200 mL with distilled water and consumed orally; the tea contained 50 mg of free gallic acid, which was found at concentrations of 0.30 μmol/L and 0.15 μmol/L in plasma and urine, respectively 50.

### Flavonoids

The bioavailability of flavonoids is typically poor, considering their health promise, and may vary significantly across various groups and individual compounds in a specific class. The relative urinary excretion of daidzein and anthocyanin has been estimated to be 43% and 0.3%, respectively, explaining the fluctuation in flavonoid bioavailability 51. Several laboratory and clinical trials are ongoing to determine the bioavailability of flavonoid compounds. In a study by Aziz et al., volunteers consumed onions containing a significant amount of conjugated quercetin (139 mg). They resulted in 1.34 mM plasma concentration and 0.8% urinary excretion 52. A single dose of pure flavonoid glycoside crystals, contained in 500 mg of naringin stirred into 240 ml water, resulted in 4.9% urinary excretion 53.

### Stilbenes

Stilbenes are a class of phenolic compounds present in different edible plants, including grapes and peanuts. Several studies have explored their bioactivity and possible benefits to human health. Stilbenes' phenolic structure (various chemical substitutes and polymerization) is highly diverse and an obstacle to understanding their metabolism and absorption rates. Among all known stilbenes, resveratrol has been a special focus of study, and results show that it has many biological functions, including anti-inflammatory, antioxidant, and antiproliferative activity 54. Researchers have moved forward with clinical and pharmacological studies to determine the bioavailability of stilbenes. A study of 500 mg of resveratrol in the human body showed a 0.28% plasma concentration 55.

### Lignans

Flaxseed is one of the richest resources of lignan and is increasingly used as a food supplement. Plant lignans may be transformed into enterodiol, enterolignans, and enterolactone by gut microbiota. Information about their bioavailability is important for careful assessment of the possible health consequences of enterolignans. Numerous studies have already been undertaken to evaluate the bioavailability of flaxseed-derived tannins. According to Bartkiene et al., consuming flaxseed at 0.3 g/kg body weight per day led to a plasma concentration of 65 nmol/L and 20 nmol/L of enterolactone and enterodiol, respectively 56.

## Polyphenols targeting the NF-κB pathway in neurological diseases

### Alzheimer's disease (AD)

AD can be identified by loss of memory, cognitive defect, and neuropsychiatric issues. The brain is damaged by amyloid-β (Aβ) deposits in neuritic plaques and hyperphosphorylated tau protein in neurofibrillary tangles. The buildup of these deposits causes NF-κB activation in both glial cells and neurons, potentially leading to protective or harmful effects 57. Polyphenols have a propensity for selectively targeting NF-κB pathway, leading to desired therapeutic effects with a favorable benefit-to-risk ratio. A research group investigates the impact of resveratrol, a naturally occurring phytoestrogen, on the loss of hippocampus neurons and memory impairment caused by amyloid-β. The study involving adult Sprague-Dawley (SD) rats displayed that Aβ administration decreased spatial memory, increased cellular concentration of iNOS, lipid peroxidation, and reduced HO-1. However, resveratrol administration enhanced spatial memory and protected against neurotoxicity caused by Aβ. The neurotherapeutic action of resveratrol was linked to a drop in lipid peroxidation levels and iNOS and an upsurge in HO-1 synthesis. The results suggest that iNOS is responsible for lipid peroxidation generated by Aβ and downregulation of HO-1. Additionally, resveratrol protects mice from neurotoxicity by inhibiting the production of iNOS 58. Another research group assessed the impact of resveratrol on oxidative cellular death in PC12 cells produced by Aβ. The cells were exposed to Aβ, leading to a notable rise in the accumulation of intracellular ROS and cell death. Aβ therapy reduced mitochondrial membrane potential, fragmented poly (ADP-ribose) polymerase, increased Bax/Bcl-XL ratio, and initiated c-JNK activation. Resveratrol mitigated these effects, while beta-amyloid enhanced NF-κB pathway activation in PC12 cells, but this effect was inhibited when pretreated with resveratrol 59. The compound LD55, a synthetic derivative of resveratrol with no hydroxyl group, reduced the formation of Aβ plaques and neuroinflammation in an AD model. This suggests that the neurotherapeutic properties resveratrol may manifest without its antioxidant properties 60. However, the trimethoxy analog of resveratrol, lacking hydroxylation, failed to offer neuroprotection against glutamate-induced cytotoxicity in neuronal cells 57,61. The administration of resveratrol and LD55 at a dosage of 0.01% by weight in the meals of transgenic mice for one year, starting from when the animals were 6 weeks old, resulted in the prevention of both microglial activation and plaque buildup in their brains. The discovery of simultaneous reductions provides evidence in favor of the hypothesis that there is a strong association between inflammation and Aβ plaque accumulation in AD. The study found that resveratrol's antioxidant properties were not the primary cause of its effects, as LD55, which lacks hydroxyl groups, showed comparable potency. The findings presented in this study provide evidence to support an alternate theory, suggesting that the decrease in neuroinflammation and plaque density could be attributable to the inhibition of NF-κB, which serves as a crucial mechanism 60.

Curcumin has anti-inflammatory properties. In addition, curcumin can effectively suppress cytokines and reduce inflammation. Curcumin also reduces pro-inflammatory cytokine production and the generation of neurotoxic factors in monocytes and alveolar macrophages 62. Curcumin can effectively inhibit the NF-κB pathway, a mechanism renowned for reducing inflammation 63,64. Its inhibitory effects include the phosphorylation and subsequent IκBα degradation and the migration of NF-κB p65 into the nucleus. The function of curcumin in alleviating memory impairments associated with AD was linked to triggering the PRAR-γ pathway, which suppresses the NF-κB pathway, leading to a decrease in the neuroinflammatory response 64. Curcumin has been found to reduce spatial memory impairments in mice with the APP/PS1 genetic mutation and positively affect cholinergic neuron function. Curcumin decreases microglia and astrocyte activation, generates cytokines, and inhibits the NF-κB signaling pathway, suggesting its potential to suppress neuroinflammation in AD. However, these benefits were diminished when PPARγ was co-administered with the GW9662 antagonist or when PPARγ gene expression was silenced. The direct binding affinity of curcumin towards PPARγ enhanced transcriptional activity and elevated protein levels. The study suggests curcumin can target PPARγ, mitigating neuroinflammation and enhancing neuronal function in AD 65.

The multifaceted molecular functions of curcumin are associated with its numerous benefits. These functions include the regulation of pathways for signal transduction and transcription factor activation. Demethoxycurcumin inhibits NF-κB activation, IkBα phosphorylation, and proinflammatory gene expression in LPS-induced microglial cells 66. In human astroglial cells stimulated by Aβ, tetrahydro-curcumin suppresses IL-1 and -2 activity, promoting neuroprotection 67.

One of the active ingredients of soybean isoflavones, genistein, is accountable for various pharmacological actions. These activities include anti-estrogen, antioxidant, and anti-inflammatory effects, along with the alteration of signaling pathways 64. A study investigated the neuroprotective properties of genistein in reducing inflammation caused by Aβ peptide 25-35 (Aβ25-35). It found that genistein mitigated the cytotoxic effects and inflammatory damage of Aβ25-35, reversed the upregulation of NF-κB and TLR4 expression, and attenuated the transcriptional functionality of NF-κB. The research also highlighted the role of NF-κB-associated pathways in this process 68. According to another research, it was shown that genistein has the potential to mitigate cell death generated by Aβ25-35 and impede the formation of TNF-α and IL-1β from C6 cells caused by Aβ25-35.

Furthermore, genistein effectually abridged the elevation of TLR4 protein and gene expression generated by Aβ25-35. Additionally, genistein demonstrated a substantial increase in IκB-α expression in C6 cells that were subjected to disruption by Aβ25-35. Genistein has the potential to mitigate the inflammatory stress caused by Aβ25-35 therapy. This effect may be linked to the neuroprotective properties of genistein, which include the modification of the NF-κB/TLR4 signaling pathway 69. The formation of inflammatory mediators caused by Aβ25-35 therapy was reduced by genistein. The administration of Aβ25-35 did not lead to any notable alterations in the expression of the mRNA that encodes for NF-κBp65.

Nonetheless, the observed increase in protein expression was mitigated by prior administration of genistein. Following the use of the siRNA approach to suppress the mRNA expression of NF-κBp65, the subsequent administration of Aβ25-35 or genistein inhibited inflammatory factor synthesis. The outcomes of this research recommend that genistein can mitigate inflammation via modulating the NF-κB pathway in C6 glial cells administered with Aβ25-35 70.

**Figure 1:**
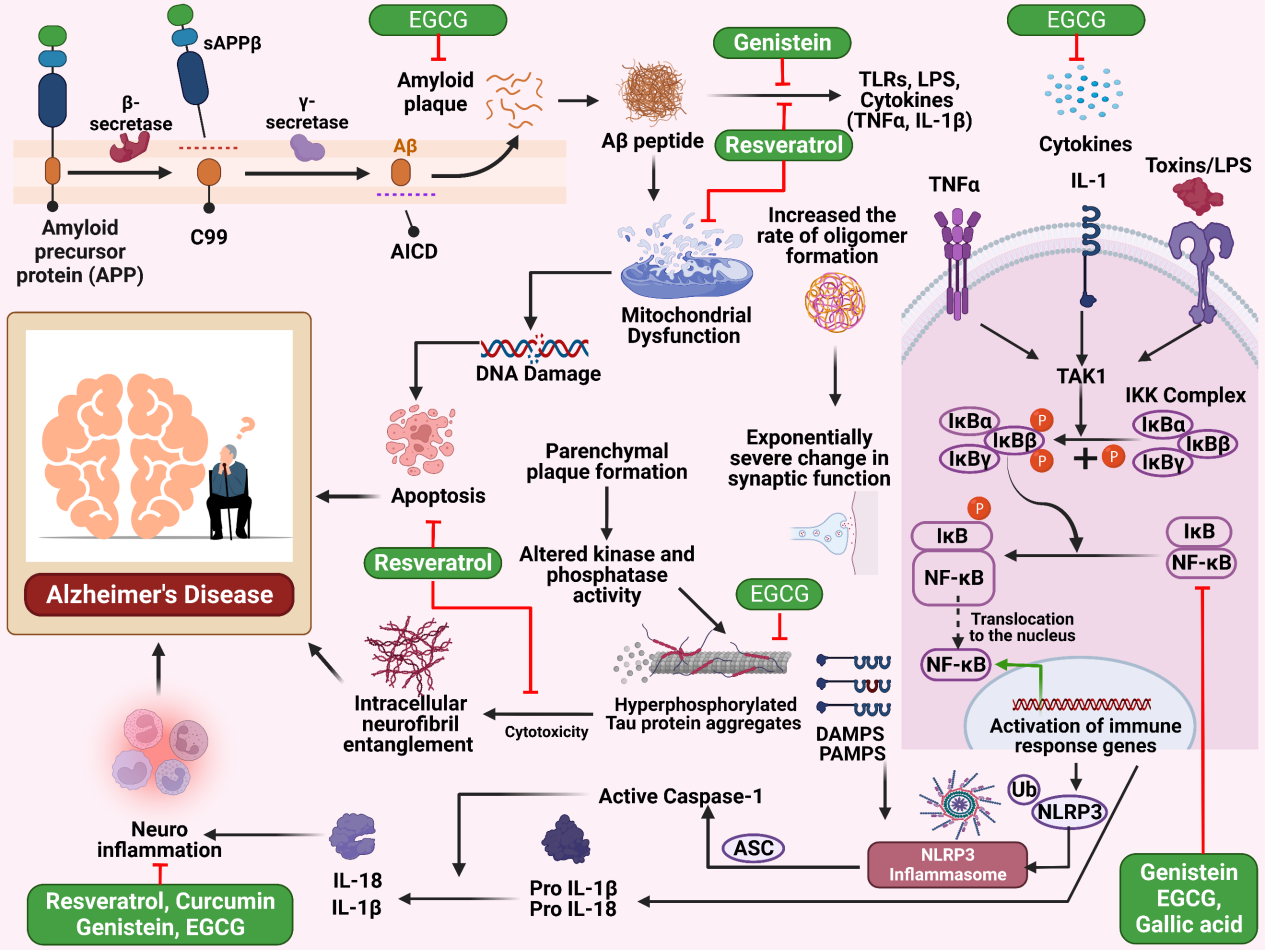
Illustration represents the polyphenols targeting the NF-κB pathway associated with Alzheimer's disease. Inappropriate breakdown of amyloid precursor protein by beta and gamma-secretase results in the creation of amyloid-beta plaque, which in turn leads to mitochondrial malfunction and apoptosis, which ultimately results in Alzheimer's disease. Cytokines and toxins also are responsible for activating the NF-κB pathway, which ultimately results in inflammation in the nervous system and ultimately leads to Alzheimer's disease state.

Gallic acid is a phenolic compound in tea leaves, gallnuts, and many other plant sources 71. A study investigated the neuroprotective benefits of gallic acid. In this experiment, gallic acid was applied to BV-2 and primary microglia cells. Gallic acid therapy effectively suppressed NF-κB acetylation, reducing cytokine generation in microglia cells. This treatment also protected neuronal cells by mitigating neurotoxicity generated by Aβ **(Figure 1)**. In mice, gallic acid demonstrated a therapeutic effect on cognitive impairment generated by Aβ. In conclusion, gallic acid effectively reserved neuronal cell death by preventing cytokine production and decreasing NF-κB acetylation level 72.

A study investigates the impact of EGCG on memory impairment in mice. The researchers administered EGCG at 1.5 and 3 mg/kg doses for 3 weeks. The mice showed a dose-dependent reduction in α-secretase levels and an increase in the brain's both β- and γ-secretase activity. EGCG also decreased the production of metabolic products derived from β- and γ-secretases. The research also revealed that EGCG effectively suppressed the stimulation of ERK and NF-κB in mice brains injected with Aβ1-42.

Furthermore, EGCG significantly inhibits apoptosis of neuronal cells induced by Aβ1-42 **(Figure 1)**. The findings suggest that EGCG may benefit from treating memory impairment 73. The simultaneous administration of EGCG and GA in the experiment did not provide any unforeseen detrimental effects in the *in vivo* setting. The potential neuroprotective and neurodegenerative effects were shown in the animal model, while the combined administration of EGCG and gallic acid demonstrated a considerable delay in illness start, a substantial reduction in severity, even after the manifestation of symptoms, and a decrease in inflammatory infiltrates. The findings demonstrated the promising benefits of integrating neuroprotective and anti-inflammatory interventions. Moreover, these results enhance the likelihood of using EGCG as a supplementary treatment option for neuroinflammatory and neurodegenerative conditions 74.

### Parkinson's disease (PD)

PD, the 2nd most prevalent ND, affects around 2% of those aged 65 and above, impacting dopaminergic neurons located in the substantia nigra (SN), which leads to a reduction in dopamine levels 40,75. Polyphenols have been extensively researched for their effectiveness in treating PD. For example, the potential role of hyperoside in alleviating microglia-mediated neuroinflammation was examined by a research group. The BV2 cells were pretreated with hyperoside compound and then activated with LPS. Hyperoside had a substantial suppressive action on generating NO and inflammatory cytokines, which LPS produced.

Additionally, hyperoside was found to suppress the expression level of iNOS. Comparable outcomes were reported in newborn mice-derived microglial cells. The findings from the analysis conducted on NF-κB and MAPK signaling, together with particular inhibitors, indicate that hyperoside can mitigate the inflammatory reactions induced by LPS via the stimulation of NF-κB and p38 pathways. In addition, hyperoside effectively inhibited the neurotoxicity mediated by activated microglia, as demonstrated by conditioned media culture. However, hyperoside did not directly influence the toxicity produced by MPP+ in SH-SY5Y neuroblastoma cells. The aggregated evidence indicates that hyperoside has the potential to function as a protective drug by mitigating the activation of microglia in neurodegenerative conditions including PD 76.

In the research directed by Sharma et al., it has been displayed that curcumin provides neuroprotection and prevents the aggregation of α-Synuclein in a PD model caused by LPS. Supplementation of curcumin effectively inhibited the activity of NF-κB protein generated by LPS. Curcumin demonstrated the capacity to interfere with the production of α-synuclein aggregation, as shown by the analysis of α-Synuclein gene and protein activity utilizing RT-PCR and immunohistochemistry, respectively. According to the study, curcumin shows promise as an option for therapy for targeted management of PD and other synucleopathies 77. A study investigated the protecting action of quercetin against Mn-induced neurotoxicity. The outcomes reported that Mn considerably reduced the survival of SK-N-MC cells, reducing lactate dehydrogenase release. However, pretreatment with quercetin at conc. 10 and 20 µg/mL mitigated this effect. Quercetin pretreatment reduced Mn-induced mitochondrial dysfunction, OS, and apoptosis. The study also found that quercetin pretreatment downregulated NF-κB, which prevented inflammatory reaction and apoptosis generated by Mn in both SD rats and SK-N-MC cells 78.

**Figure 2:**
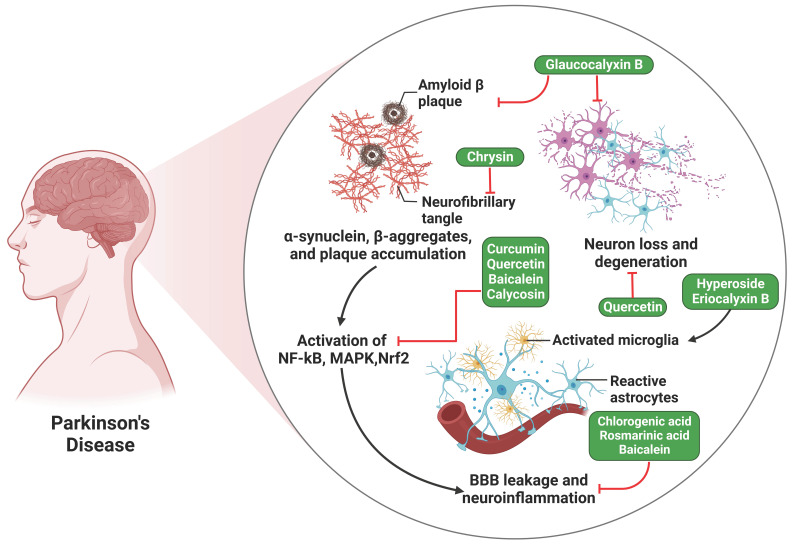
Illustration represents the polyphenols targeting the NF-κB pathway associated with Parkinson's disease. The activation of the NF-κB, MAPK, and Nrf2 pathways by amyloid-beta plaque and neurofibrillary tangles ultimately results in the leaking of the blood-brain barrier (BBB) and neuroinflammation, which ultimately leads to Parkinson's disease.

A study examined the effects of chlorogenic acid (CGA) supplementation on mice intoxicated with MPTP. It assessed glial cell activation and OS levels in the SN and striatum. Results showed that CGA effectively inhibited neuroinflammation by modulating NF-κB expression, suppressing pro-inflammatory mediators, including IL-1β and TNF-α, and promoting anti-inflammatory cytokine production (IL-10). Additionally, CGA decreased GFAP staining in the SN area, indicating its therapeutic potential in lowering OS and neuroinflammation in mice. The findings suggest that CGA supplementation may have therapeutic potential in reducing neuroinflammation and OS in mice **(Figure 2)** 79. Besides, another study found that rosmarinic acid administration improved motor performance in mice with PD.

Furthermore, it resulted in an augmentation of tyrosine hydroxylase-positive cells, a reduction in the formation of inflammatory cytokines, and a suppression of microglia activation. Rosmarinic acid also reduced pro-inflammatory cytokine release from MPP+ or α-synuclein. This suggests that rosmarinic acid can reduce neuroinflammation by inhibiting the TLR4/NF-κB/HMGB1 pathway, potentially demonstrating its anti-PD efficacy **(Figure 2)** 80.

A separate research endeavor examined the consequences of baicalein in rats with PD caused by rotenone, emphasizing its anti-inflammatory action and investigating the underlying processes. The findings from the study demonstrated that baicalein administration improved motor function, ameliorated brain injury, mitigated proinflammatory cytokines, regulated astrocyte and microglia stimulation, and restricted NF-κB signaling pathway activation. Baicalein also inhibited the formation of NO and iNOS proteins by impeding the phosphorylation of NF-κB and IκBα. Additionally, baicalein downregulated the TLR4, a fundamental part of the NF-κB pathway in activated BV2 microglia. The research suggests baicalein has potential efficacy in addressing PD se due to its anti-neuroinflammatory properties 81. A research investigation by Xu et al. documented that glaucocalyxin B has a mitigating effect on PD caused by LPS through the suppression of the TLR/NF-κB pathway and stimulation of the HO-1/Nrf2 pathway. PD developed in laboratory rats by administering LPS into the cerebral corpus striatum. Subsequently, the animals were treated with GLB, which was administered intracerebroventricularly (ICV).

The administration of LPS resulted in locomotor impairments, such as walking and climbing difficulties, and sensory problems in rats. Inflammation, OS, and apoptosis accompanied this LPS-induced reaction in the cerebral tissue. Furthermore, the injection of LPS stimulated the NF-κB/TLR signaling pathway and the Nrf2/HO-1 pathway in the cerebral tissue. The administration of glaucocalyxin B reduced LPS-induced changes. Following the administration of glaucocalyxin B, the NF-κB/TLR signaling pathway was rendered inactive, whereas the Nrf2/HO-1 pathway was stimulated. The cytotoxic action elicited by the conditioned media obtained from microglial cells subjected to LPS was mitigated by glaucocalyxin Bin PC12 cells 82. Another study has demonstrated that in mice models of PD, the compound eriocalyxin B has shown its ability to safeguard dopaminergic neurons via inhibiting microglia activation. This leads to improved motor dysfunction, alleviated inflammation, and ameliorated dopamine neuron degeneration. Eriocalyxin B effectively mitigates the negative effects of microglia activation generated by MPP+ on dopamine neurons. It reduces proinflammatory cytokine production in microglia through phenotypic alteration of microglial cells and activates transcription and NF-κB signaling pathways. This study suggests that eriocalyxin B has strong anti-inflammatory properties by selectively influencing microglia activation, protecting against harm to dopamine neurons produced by MPP+. This research could offer valuable insights into the possible medical applications of Eriocalyxin B in PD 83. Another experimental study explored the potential of calycosin in mitigating PD symptoms in mice. The disease was produced through injection of MPTP and then treated with calycosin ICV. Immunostaining techniques were used to determine dopaminergic neurons and microglia statuses. In the research, qPCR was used to measure inflammatory components in the sample. Western blot analysis examined the contributory role of NF-κB/TLR and MAPK pathways in the pathogenesis of PD. The study found that calycosin effectively alleviated behavioral impairments and inflammatory reactions in mice with MPTP-induced PD. A pioneering study has suggested that calycosin has a mitigating effect on symptoms of PD by modifying the NF-κB/TLR pathways in both mice models and cellular cultures 84.

To observe the effect of hesperetin on symptoms resembling PD, a 50 mg/kg dose of hesperetin was administered for one week. Based on the results obtained, it was shown that hesperetin exhibited a reduction in OS via the modulation of the Nrf2, attenuation of neuroinflammation mediated by NF-κB, and mitigation of apoptotic cell loss. Furthermore, it was shown that hesperetin significantly attenuated motor impairment in rats with PD produced by 6-OHDA 85. A separate research was performed to examine the potential role of hesperetin on neuroinflammation and neurodegeneration generated by Aβ. The cumulative outcomes of the research showed that hesperetin mitigated Aβ-induced OS, reducing the activation of microglial cells and astrocytes. The inhibition of glial cells was concomitant with a decrease in NF-κB phosphorylation and the secretion of inflammatory mediators. The neuroprotective benefits of hesperetin against neuroinflammation generated by Aβ were also validated by *in vitro* investigations, which demonstrated that the suppression of p-NF-κB and TLR4 by hesperetin was equivalent to those of the particular inhibitor p-NF-κB 86. A separate study involved administering LPS to mice and evaluating their anti-inflammatory effects post-administration. The results demonstrated that the injection of hesperetin led to a noteworthy decline in the inflammatory reaction, as shown by the decreased expression of p-NF-κB. The researchers conducted a comparative analysis of the suppressive actions of several pharmacological inhibitors on TLR and phosphorylated NF-κB. The overall results of their study supported the claim that hesperetin effectively mitigated the inflammation generated by LPS 87,88. Chrysin, a flavonoid compound found in honey, bee propolis, and other plant sources, has been found to have a neurotherapeutic potential in mice models. Nevertheless, further investigation is required to comprehend the precise function of neurotrophic factors, inflammatory cytokines, and neuronal recovery in its neuroprotective effects. The study found that administering 6-OHDA by microinjection led to changes in mouse behavior, increased levels of cytokines, and a drop-in interleukin-10 level. Oral administration of chrysin mitigated these changes, confirming its neuroprotective properties in PD treatment **(Table 1)** 89.

Cerebral ischemia is a significant global health concern, with stroke costs predicted to reach $240.67 billion by 2030. Current treatments for this condition are limited, with only anti-thrombolytics and hypothermia being effective. This highlights the need for innovative interventions to address stroke and cerebral ischemia. Resveratrol has shown promise in ameliorating subarachnoid hemorrhage, highlighting the urgent need for innovative interventions 113. Research was directed to evaluate the potency of resveratrol, a phenol known for its antioxidant properties, on BV2 microglial cells in a hypoxic damage model. The mRNA expression of pro-inflammatory TNF-α was inhibited by resveratrol, while the mRNA expression of anti-inflammatory IL-10 was enhanced in BV2 microglia. Resveratrol, a compound, was found to regulate inflammatory responses in BV2 microglia, which are crucial in regulating inflammatory responses in hypoxia-induced injury. Additionally, the application of resveratrol was displayed to increase the upregulation of BDNF in BV2 microglia subjected to hypoxic conditions. In general, it is recommended that resveratrol has the potential to enhance the advantageous role of microglia in the context of ischemic brain injury 114. In a separate investigation, the stimulation of the NF-κB pathway was observed via the IκB phosphorylation and the subsequent translocation of NF-κB p65 into the nucleus. According to the findings, the administration of quercetin prior to therapy exhibited a notable enhancement in cardiac function, reduced myocardial damage, and decreased infarct size. Quercetin therapy has significantly improved in both *in vivo* and *in vitro* scenarios, effectively mitigating myocardium oxidative damage and apoptosis. The effects of quercetin during myocardial ischemia-reperfusion injury (IRI) were seen to be associated with the suppression of the NF-κB pathway activation. However, when PPARγ or GW9662 knockdown was introduced, the cardioprotective benefits of quercetin were only partly diminished. In summary, the findings reported that the administration of quercetin effectively mitigated heart damage generated by IRI via the activation of PPARγ. The underlying mechanism may entail the NF-κB pathway suppression through the activation of PPARγ 115.

A research group explored the pharmacological action of naringenin on mitigating OS and reducing inflammatory damage to the brain in a rat model of focal IRI. Naringenin was given daily to Wistar rats for 21 days, followed by middle cerebral artery blockage and reperfusion. The administration of naringenin enhanced antioxidant capacity, reduced infarct size, and decreased levels of NO, myeloperoxidase, and cytokines. It also improved functional recovery levels. Immunohistochemistry and Western blot investigations showed that naringenin inhibited glial stimulation and decreased NF-κB expression. The study concluded that naringenin as a preventive measure improved functional outcomes and mitigated damage caused by ischemic brain injury by inhibiting NF-κB-mediated neuroinflammation. The findings mentioned above indicate that naringenin has potential as a neurotherapeutic compound for those with a greater vulnerability to ischemic stroke 90. The preventive effects of genistein against cerebral ischemia in a mice model were investigated. Mice were given genistein orally at 2.5 to 10mg/kg doses for 14 consecutive days before the induction of temporary MCAO. The study found that genistein reduced infarct volume, developed neurological damage, and stopped cell death after ischemic events. It also suppressed the generation of ROS produced by ischemia and improved the enzymatic activities of glutathione peroxidase and SOD. Additionally, genistein reduced malondialdehyde levels in stroke-affected mice. Genistein restored normal mitochondrial function after ischemia, stopping cytochrome C release, reducing ROS level, and preventing activation of CASP3. Western blotting analysis showed that Ischemia triggered NF-κB signaling activation by ROS. Genistein treatment also decreased the phosphorylation and degradation of IκBα. The study suggests that genistein may prevent brain damage caused by transient localized ischemia, which is likely mediated via modulation of mitochondria-induced apoptotic pathways and inhibition of ROS-generated NF-κB stimulation 91.

Catechin hydrate (CH) has been shown to help mitigate redox imbalance and reduce inflammation in cases of focal cerebral ischemia. A study on male Wistar rats displayed that CH pretreatment resulted in a noteworthy decrease in infarct size, neurological impairments, neuronal death suppression, and downregulation of iNOS and NF-κB expression in the ischemic brain. The MCAO group showed a reduction in antioxidant enzyme activity and glutathione levels, but this was considerably mitigated in the CH pretreatment group. The MCAO group also increased thiobarbituric acid reactive species and cytokines, while CH pretreatment reduced these markers. The study suggests that CH protects the brain against damage induced by MCAO, likely due to the downregulation of NF-kB expression. This recommends that CH could be a potential candidate for focal cerebral ischemia treatment 92. Another study looked at how flavonoid luteoloside protects against cerebral ischemia. The research involved male Sprague-Dawley rats exposed to MCAO to examine the role of luteolysis on IRI. The findings demonstrated that luteoloside improved cerebral infarction, mitigated neurological impairments and cerebral edema, and reduced neuroinflammation. The administration of luteoloside also inhibited the NF-κB activation, upregulated the protein expression of PPARγ, and increased the nuclear accumulation of Nrf2 in the brain tissues of rats subjected to MCAO. The study concluded that luteoloside has a significant neuroprotective effect by suppressing NF-κB activation in a rat ischemia model. The involvement of PPARγ and Nrf2 in the anti-inflammatory effect of luteoloside was also significant. The outcomes reported that luteoloside has potential efficacy in treating cerebral ischemia and various NDs 93.

Furthermore, several research investigations have shown the effect of mangiferin on the modulation of NF-κB pathway and mitochondria-associated processes, as well as its influence on the NLRP3 inflammasome, an essential element of the innate immune system 116. Based on research conducted by Marquez et al., mangiferin possessed anti-inflammatory and antioxidant properties in rats subjected to a stress-induced paradigm, reducing inflammation and mitigating oxidative damage. Additionally, the authors observed that MFN targeted many factors, including lipid peroxidation, catalase activity, elevated levels of pro-inflammatory mediators, and enhanced NF-κB activation 117. Additionally, kaempferol-7-O-rutinoside was found to have neuroprotective, anti-inflammatory, and anti-apoptotic properties by inhibiting phosphorylation of NF-κB p65, reducing COX-2, ICAM-1, iNOS, BAX2, and suppressing CASP3 and CASP9 cleavage 118,119.

### Anxiety and depression

Depression and anxiety are common mental disorders with complex causes at the intersection of various biological systems. They cause various physical and mental symptoms, such as irritation, impatience, fatigue, and difficulty focusing. Each manifestation requires a unique treatment approach. The serotonergic and noradrenergic systems are involved in mood disorders, including anxiety and depression. The serotonergic system regulates mood and hunger while impacting cognitive processes in the brain. Treatment for these disorders requires a unique approach to each symptom among individual 120.

Wei et al. conducted a study that demonstrated the potential of resveratrol in mitigating depression- and anxiety-associated behaviors produced by maternal separation. The researchers also observed a reduction in neuroinflammation mediated by the Sirt1-NF-κB signaling pathway. The study found that maternal separation from their children increased anxiety and sadness, pro-inflammatory cytokines, and suppressed the activity of the NF-κB/Sirt1 signaling pathway in male offspring. However, resveratrol administration was found to counteract these effects and restore normal functioning. The effect was likely mediated via the activation of the NF-κB/Sirt1 pathway, suggesting that resveratrol may alleviate depression- and anxiety-dependent symptoms 94.

A study found that resveratrol has antidepressant properties, as inflammation, OS, and apoptosis may contribute to the progression of depression. Exposure to chronic unpredictable mild stress for 8 weeks led to reduced expressions of β-catenin, GSH, Bcl-2, and total antioxidants, while increased expressions of NF-κB, GSK-3β, IL-1β, TNF-α, and MDA in the hippocampus. Resveratrol administration improved the condition by mitigating neuroinflammation, OS, and apoptosis while increasing concentrations of BDNF and β-catenin in the brain 95. Another research evaluated the activity of resveratrol to mitigate depression- and anxiety-associated symptoms generated by ovariectomy in mice. The use of resveratrol led to a reduction in depressive and anxious behaviors in mice. This effect was achieved by modulating microglia activation and downregulating NLRP3 and NF-κB signaling pathways in the hippocampus. Furthermore, resveratrol supplementation elevates Sirt1 levels, contributing to its therapeutic effects. The authors of this study have determined that resveratrol can improve the psycho-behavioral changes caused by estrogen shortage by suppressing inflammatory pathways. Additionally, they found that resveratrol is useful in mitigating postmenopausal modifications 96,121.

The administration of proanthocyanidin has been shown to effectively mitigate the progress of depressive-like activities in mice that have been exposed to lipopolysaccharide. This beneficial effect is believed to occur via the modulation of the neuroinflammatory system. The upregulation of COX-2 and iNOS caused by LPS was decreased by proanthocyanidin. This inhibition occurred by modifying NF-κB in the prefrontal cortex (PFC), hippocampus, and amygdala. When considering the collective evidence, it can be concluded that proanthocyanidin exhibits potential as a therapeutic intervention for depression-like behavior generated by LPS via its notable anti-inflammatory characteristics 97. Besides, the administration of curcumin has demonstrated the ability to mitigate depressive-like characteristics in rats exposed to a persistent regimen of unexpected moderate stress. This effect is mediated via the suppression of the NLRP3 inflammasome. Curcumin has been shown to exhibit a significant reduction in the mRNA expression of proinflammatory cytokines, including TNF-α, IL-1β, and IL-6, while also demonstrating the ability to inhibit the activation of NF-κB. In addition to its inhibitory effects on the activation of the stressed-induced P2X7R/NLRP3 inflammasome axis, curcumin also demonstrated a reduction in the change of pro-IL-1β to mature IL-1β. Curcumin supplementation alleviated the stress-associated stimulation of indolamine-2 and 3-dioxygenase and the subsequent rise in the kynurenine/tryptophan ratio. In summary, this research indicate that curcumin can alleviate symptoms associated with depression by inhibiting the kynurenine pathway and NLRP3 overactivation inflammasome 98.

### Spinal cord injury (SCI)

SCI is a debilitating neurological disorder characterized by a risk for paralysis and incontinence. Factors contributing to limited spinal cord axon regeneration include decreased growth of adult projection neurons, inhibitory signals from damaged myelin, glial scar formation by local astrocytes, and lack of nerve growth and neurotrophic factors. Both medical and physical treatments have no therapeutic effect 122.

Understanding the mechanisms of cell death and inflammation after SCI is crucial for developing effective therapies. NF-κB and MAPK are key signaling pathways involved in these processes, which can promote the expression of pro-inflammatory genes and lead to secondary tissue damage 123,124. Novel avenues for polyphenol treatment include developing targeted derivatives, combining polyphenols with other therapeutic agents, and encapsulating polyphenols in nanoparticles for improved bioavailability and targeted delivery 125,126. However, challenges including limited bioavailability and blood-brain barrier permeability must be addressed. More clinical trials are needed to evaluate the safety and efficacy of polyphenols for SCI treatment. Understanding the roles of NF-κB and MAPK signaling pathways in SCI can lead to novel therapeutic avenues for SCI treatment 127. The existing therapeutic approaches include the administration of high-dose methylprednisolone sodium succinate, corticosteroid medications, surgical procedures, comprehensive multisystem medical therapies, and rehabilitation interventions. Nevertheless, it is essential to explore innovative methodologies for the treatment and restoration of SCI in order to enhance the overall well-being and quality of life for affected individuals 128. SCI can impair blood vessel formation, hindering tissue repair and regeneration 129. Polyphenols, natural compounds found in plants, fruits, and vegetables, have emerged as promising therapeutic agents for SCI due to their potential to promote angiogenesis 130. These compounds have antioxidant and anti-inflammatory properties, scavenging free radicals and modulating inflammatory pathways. They can increase nitric oxide production, a key signaling molecule for angiogenesis, and stimulate the expression of vascular endothelial growth factor (VEGF). Potential benefits of SCI treatment include improved blood supply, reduced inflammation, scar tissue formation, and neuroprotection 131.

Research was performed to assess the impact of oleuropein aglycone, a derivative of oleuropein hydrolysis, on the inflammatory response in mice, particularly after spinal cord injuries. Vascular clips created the lesion during a laminectomy. The study found that oleuropein aglycone administration reduced multiple inflammatory responses, including histological damage, NF-κB expression, motor recovery, protein kinase Aβ activity, pro-inflammatory cytokines, neutrophil infiltration, lipid peroxidation, nitrotyrosine formation, and apoptosis. The research suggests that phenolic components of olive oil, like oleuropein aglycone, could be beneficial in treating various inflammatory conditions 99.

The nuclear factor, a protein in molecular biology, is crucial in regulating the NF-κB pathway and is responsible for inflammation, damage from free radicals, and apoptosis in SCI. Resveratrol, a molecule with numerous health benefits, was assessed for its therapeutic potential in several organ disorders. It has antioxidant, anti-inflammatory, and anti-apoptotic properties. Resveratrol can maintain tissue structural integrity, protect neurons from apoptosis, and improve the locomotor function of hindlimb motor neurons. It also enhances the regeneration of damaged spinal cord tissues by modulating cytokine activity. Resveratrol can hinder oxidation by enhancing superoxide dismutase activity and reducing malondialdehyde levels, thereby removing harmful free radicals and protecting cellular structures. It can also mitigate myeloperoxidase activity and other inflammatory factors, reducing inflammation and decreasing neuronal cell death. Additionally, it can mitigate apoptosis by upregulating the expression of Bcl-2 and preventing Bax and CASP3, attenuating apoptosis and decreasing neuronal cell death 100,132. Resveratrol, a potent antioxidant, has been shown to improve SCI treatment outcomes by inhibiting NF-κB activity through the SIRT1-AMPK pathway. This pathway links autophagy and inflammation. Resveratrol's polydatin glucoside has antioxidative properties and could progress transplanted bone marrow stem cells' therapeutic effectiveness. It also mitigates inflammatory reactions in rats' by reducing pro-inflammatory factors, up-regulating anti-inflammatory cytokines and SIRT1, and suppressing NF-κB activity. Resveratrol also affects bone density, reducing inflammation and restoring Wnt/β-catenin and insulin-like growth factors-1 signaling pathways. Overall, resveratrol's antioxidant properties have potential applications in SCI treatment 103,132,133.

Eugenol led to noteworthy improvements in locomotor function and relief from neuropathic pain. These improvements were followed by reductions in inflammation, OS, and the presence of molecules associated with neuronal death in both the serum and the damaged spinal cord. The molecules NF-κB and p38 MAPK, associated with downregulation of pathways, were also identified in the spinal cord. This study's results indicate that Eugenol's neuroprotective properties against traumatic SCI may be facilitated by the down-regulation of NF-κB pathways 104.

**Figure 3:**
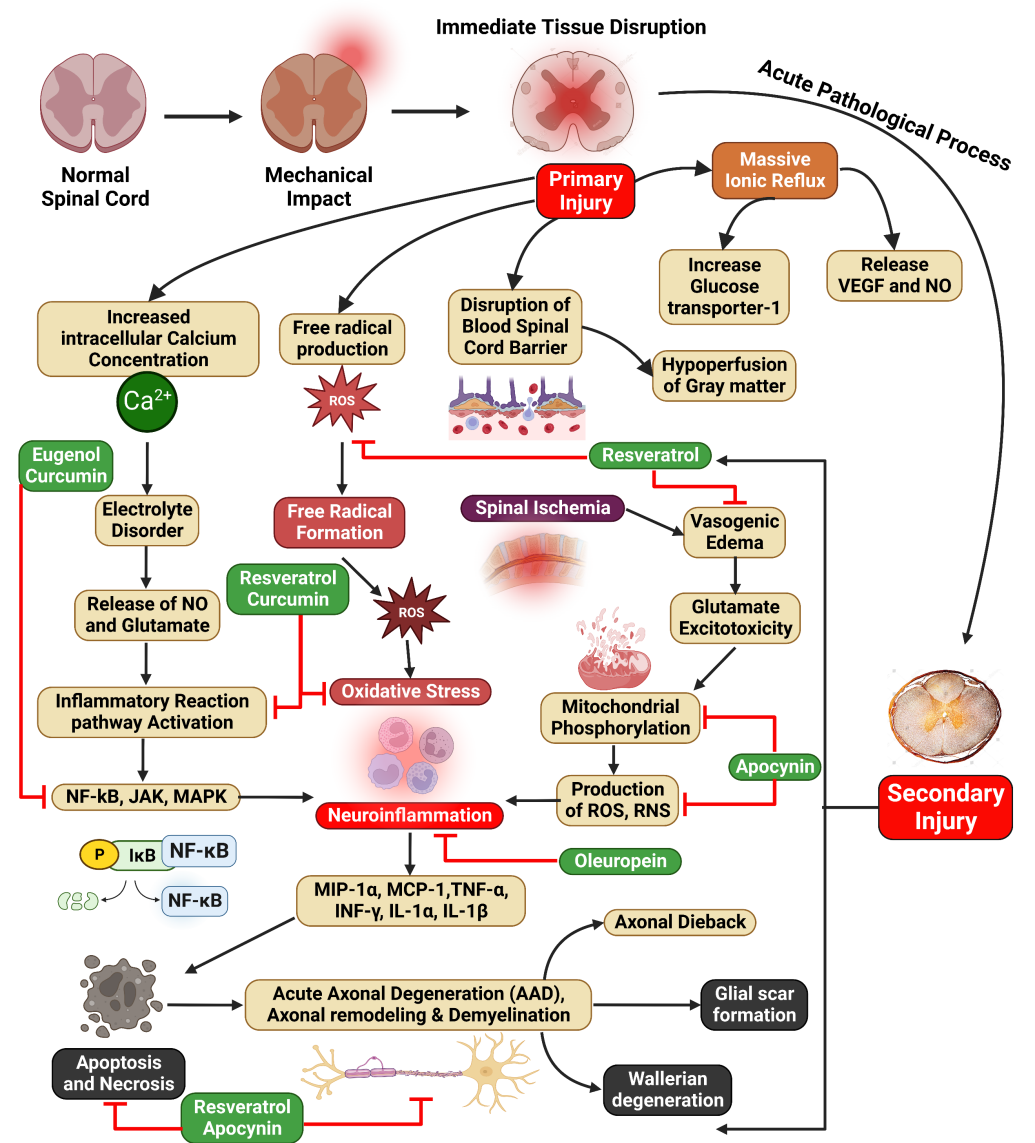
Illustration represents the polyphenols targeting the NF-κB pathway associated with SCI. SCI is characterized by primary injury followed by secondary injury. Increased intracellular calcium ion concentration, free radical production, blood-spinal cord barrier disruption, and mass ion reflux result from primary injuries, leading to the progression of secondary injuries. In addition, spinal ischemia, oxidative stress, and neuroinflammation lead to the progression of secondary SCI.

The activation of NF-κB was inhibited by curcumin, which decreased production of COX-2, IL-1, -6, -8, and TNF-α 134. Additionally, curcumin was shown to enhance the activity of SOD 105. The anti-inflammatory action of curcumin after SCI was linked to the suppression of NF-κB, IL-1β, -6, and TNF-α activity and elevation of Nrf2 106,107. Curcumin has been shown to elicit an antioxidative effect by triggering the Nrf2 pathways and subsequently reducing the levels of ROS via NF-κB activation 135. According to a study by Yuan et al., curcumin has shown a significant inhibitory effect on TGF-β, serving as an apoptotic receptor upstream of SCI. The researchers also discovered that curcumin inhibits NF-κB in both apoptotic and inflammatory processes **(Figure 3)** 136.

A study was conducted to assess the effectiveness of apocynin therapy after SCI in rats and its potential therapeutic benefits for the healing of acute damage to the spinal cord. A rodent SCI model was created, and apocynin was supplied intraperitoneally at 50 mg/kg. The first dose was given 30 minutes after the SCI, followed by further doses every 12 hours for 3 days. The results demonstrated that using apocynin led to a reduction in oxidative damage, mitigation of neuronal apoptosis, suppression of the inflammatory response, and improvement in locomotor activity. Apocynin, administered intraperitoneally at a dose of 5 mg/kg, elicited a noteworthy decrease in FasL and NF-κB pathway activation. Consequently, apoptosis following SCI was suppressed. Hence, the investigation substantiated the therapeutic effectiveness of apocynin in the treatment of SCI, likely achieved by the suppression of apoptosis and the inflammatory reaction, thereby facilitating the recovery of neural functionality **(Figure 3)** 108. Liu et al. found that apocynin can improve histology results and aid forelimb motor function recovery following SCI. The study also demonstrated its suppressive effects on various pathways, aiming to mitigate inflammatory, oxidative, and apoptotic effects post-injury 137.

### Autism spectrum disorder (ASD)

ASD is a neurodevelopmental condition distinguished by impairments in interpersonal interaction and the manifestation of limited interests and recurrent behaviors 138. According to the WHO, the estimated global prevalence of ASD is 0.76%. Notably, this figure represents only around 16% of the total global child population 139. According to the CDC, around 1.68% of children in the US who are 8 years old, or 1 in 59 children, get a diagnosis of ASD 140,141. In 2016, the US experienced a slightly higher prevalence of parent-reported ASD diagnoses, with an average of 2.5% 138,142.

ASD is a complex neurological condition with no current effective therapeutic intervention. Various experimental and clinical investigations have explored the administration of dietary polyphenols to treat key symptoms, which have diverse effects and operate through different molecular processes 143. Dietary polyphenols, a class of bioactive substances, have shown promise to prevent and treat several chronic diseases due to their ability to regulate inflammation and OS signaling pathways. Although some studies question their effectiveness in living organisms, evidence suggests they accumulate in the gastrointestinal tract, potentially affecting intestinal and neurological disorders. The "gut-brain axis" modulation may be crucial in treating ASD, a neurological condition with no clinically proven therapy. Various experimental and clinical studies have explored the use of dietary polyphenols to treat key symptoms associated with ASD, focusing on their diverse effects and molecular processes 144. The study of Figueira et al. found that blackberry polyphenols, including anthocyanins and quercetin, can substantially cross the BBB and produce remarkable neuroprotective properties. They inhibit NF-κB translocation to the nucleus and reduce the TNF-α production, demonstrating their potential for treating various health conditions 144,145.

Numerous experimental investigations have consistently shown that flavonoids and their subclasses have been found to modulate biochemical signaling pathways linked to antioxidant systems, mitochondrial function improvement, and neuroinflammation suppression. Recent research suggests that flavonoids are implicated in the regulation of key pathways, including the NF-κB, JAK/STAT, TLR, and CREB pathways, which are implicated in regulating neuroinflammation, which further induces the pathogenesis and progression of psychiatric and neurological disorders, including ASD 146,147. By using a VPA model of autism, a study explored the effects of luteolin and palmitoyl ethanolamine on autistic-like behavior alterations. The intervention reduced proinflammatory molecules like IL-1β, NF-κB, and TNF-α, influenced apoptosis markers in the hippocampus and cerebellum, and promoted enhanced neuroplasticity and neurogenesis. The VPA model was used to investigate these effects 110,147.

**Figure 4:**
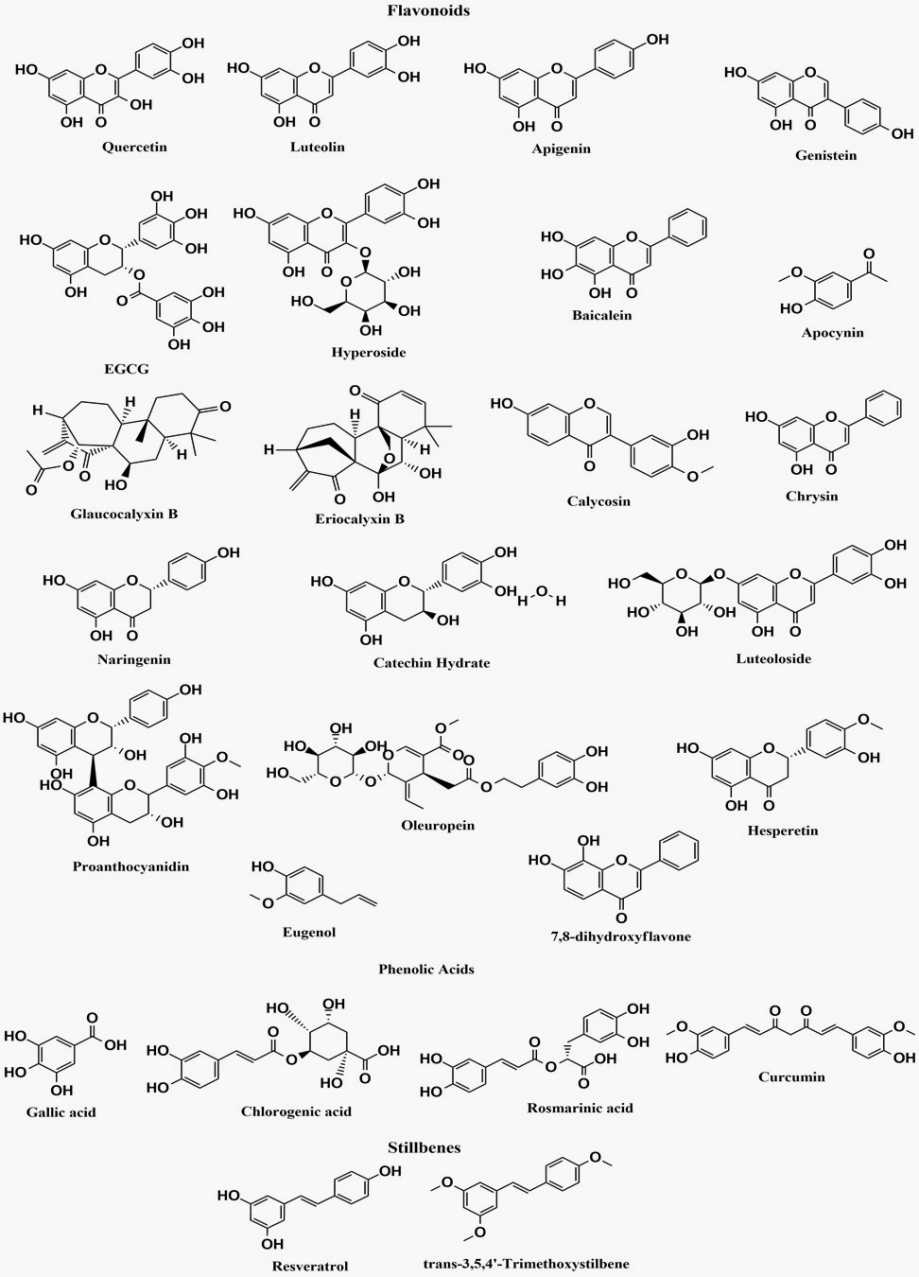
Structures of bioactive polyphenolic compounds effective in NF-κB signaling pathway-associated neurological disorders

The bioactive polyphenolic compounds that are effective in NF-κB signaling pathway-associated neurological disorders are demonstrated in **Figure 4**.

### Huntington's disease (HD)

Huntington's disease (HD) is a severe neurological condition marked by gradual deterioration of motor function, reduction in cognitive abilities, and alterations in behavior. The condition arises from a genetic mutation in the huntingtin (HTT) gene, resulting in the buildup of misfolded HTT protein in neurons. The protein aggregation process initiates many pathogenic mechanisms, including inflammation and oxidative stress, which are partly regulated by the NF-κB signaling pathway 148,149. Polyphenols are natural compounds in various vegetables, fruits, and drinks such as coffee and tea. Due to their powerful antioxidant and anti-inflammatory effects, they are considered promising options for treating neurodegenerative illnesses such as HD. Multiple studies have demonstrated that polyphenols can specifically affect the NF-κB pathway and provide neural protection in HD models 150,151. Research indicates that targeting ERK activity might be a promising approach for treating HD. The efficacy of fisetin, a polyphenol that triggers the Ras-ERK cascade, was evaluated in three Huntington's disease models. The findings demonstrated that fisetin mitigated the effects of mutant huntingtin in various experimental models. Resveratrol, a polyphenol that is closely related, also stimulated ERK and showed a protective effect in models of HD. The activation of ERK by fisetin, resveratrol, and similar drugs suggests their potential use in the treatment of HD 152. Grape seed polyphenolic extract (GSPE), a naturally occurring substance, has shown the ability to disrupt the formation of protein aggregations associated with neurological diseases. The research discovered that treatment with GSPE effectively suppressed the formation of polyQ aggregates in a PC-12 cell line. Mice given GSPE demonstrated enhanced lifespans in feasibility trials conducted *in vivo*. Treating 100 mg/kg/day GSPE orally reduced the decline in motor skills and increased the longevity of R6/2 mice. These investigations indicate that GSPE may influence the start and course of HD 153.

### Diabetic neuropathy

Polyphenols and their derivatives, plentiful in vegetables, fruits, and certain teas, have become potential therapeutic agents for relieving diabetic neuropathy. These powerful antioxidants help fight against the oxidative stress characteristic of diabetes, preserving nerve cells from harm caused by free radicals 154. Polyphenols such as resveratrol, curcumin, and EGCG might prevent or postpone the development of symptoms associated with diabetic neuropathy, such as numbness, tingling, and pain, by scavenging ROS and enhancing antioxidant defenses. Moreover, their anti-inflammatory effects might mitigate nerve damage from persistent inflammation linked to diabetes 155. In addition, some polyphenols, such as EGCG, have shown the ability to stimulate the regrowth of nerves, which may assist in restoring injured nerve fibers 156.

Research exploring the possible benefits of luteolin for diabetic neuropathy discovered that it relieved atypical feelings, enhanced nerve conduction velocities, and increased blood flow in diabetic rats. Luteolin exhibited a dose-dependent reduction in the formation of ROS, malondialdehyde levels, and a rise in antioxidant activity. In addition, it increased the expression of Nrf2 and HO-1 proteins in neurons affected by diabetes. The results emphasize the therapeutic efficacy of luteolin in enhancing diabetic neuropathy, a significant contributor to illness in individuals with diabetes. The research highlights the potential of luteolin as a therapy for diabetic neuropathy 157. Curcumin, a biologically active compound found in turmeric, has shown therapeutic properties in various medical conditions. However, its impact on diabetic peripheral neuropathy (DPN) remains insufficiently investigated. An experiment was carried out using rats to investigate curcumin's therapeutic advantages and mechanisms in diets rich in fat and sugar. The rats were categorized into five groups, and the findings indicated that curcumin did not have a noteworthy impact on body weight and fasting blood glucose. Nevertheless, curcumin administered at 100 and 150mg/kg doses reduced the mechanical withdrawal threshold, nerve conduction velocity, and sciatic nerve ultrastructure in rats with DPN. Furthermore, it decreased programmed cell death in the sciatic nerves of Schwann cells. The research found that curcumin has neuroprotective properties for treating DPN 158.

## Updates on clinical status

The available epidemiological evidence indicates that regular consumption of citrus fruits has been linked to positive health outcomes, for example, a decreased likelihood of cerebral infarction, cardiovascular disease, and ischemic stroke. However, there is a need to investigate the immediate impact of consuming beverages high in flavonoids on cognitive performance in the post-meal interval. In a study involving individuals aged 30 to 65, 240 ml of FR orange juice with 272 mg of a substance and a placebo with the same number of calories were given. The order of consumption was randomly determined, and the study assessed cognitive performance and subjective mood at baseline, before ingestion, and 2 and 6 hours after consumption. The outcomes indicated a substantial improvement in executive function and psychomotor speed test scores after consuming the FR drink compared to the placebo. The study also provided evidence for the positive impact of objective cognitive performance on subjective alertness, with considerable improvements seen in those who consumed the FR drink compared to those who received the placebo. The effect was observed to last for six hours 159. The research was conducted using a double-blind, randomized, and controlled design to examine the impact of 336.4 mg of decaffeinated green tea catechins (GTC) on cognitive performance. The investigation assessed the effects immediately after a single dose and over 12 weeks of daily consumption. The participants in this research consisted of Japanese people aged 50 to 69 years who had a Mini-Mental State Examination Japanese version score of more than 24 and reported experiencing cognitive deterioration. The researchers used the Cognitrax testing battery to assess cognitive functioning. After administering a single dosage of GTC, there was a considerable drop in the wrong answer rate on the Continuous Performance Test. Following 12 weeks during which participants consumed GTC daily, a notable reduction in reaction time was seen specifically in Part 4 of the 4-part Continuous Performance Test. Part 4 of this test involves a two-back task. The findings of this study indicate that the regular use of GTC may potentially provide positive outcomes on cognitive function, specifically concerning working memory 160.

The ingestion of cocoa flavanols (CF) has been shown to favorably impact certain physiological processes, indicating that it may potentially enhance some elements of cognitive performance. A research group aimed to examine the immediate cognitive and subjective impacts of consuming CF in prolonged mental exertion. This study used a randomized, controlled, double-blinded, balanced, three-period crossover design to investigate the effects of different doses of CF (520 mg and 994 mg) on a group of 30 healthy adult participants. The study also included a control group that took a matching control drink. To minimize any potential carryover effects, a three-day washout interval was implemented between the consumption of each drink. The 520 mg CF beverage ingestion was shown to considerably reduce self-reported 'mental fatigue' increases 161. Regular intake of flavonoids can recover cognitive performance in people with moderate cognitive impairment or neurodegenerative illness. However, no studies were performed on healthy adults or the impact of flavanones in orange juice on cognitive performance. A study found that an eight-week juice diet high in flavanones considerably improved global cognitive performance compared to a low-flavanone diet 162.

A small number of researchers have explored the use of dietary polyphenols as a new therapy for ASD. Taliou et al. conducted an open-label pilot study on 50 children with ASD who were given quercetin, luteolin, and rutin. The children were monitored for six months, and the recommended daily dosage was one capsule for every 10 kg of body weight for 26 weeks. The study found that the polyphenolic medication increased children's communication, focus, and collaboration skills while reducing aberrant behaviors. Although the research has shortcomings, the findings suggest that these bioactive chemicals may have therapeutic potential in treating ASD 163. A recent study found that children who received a polyphenolic formulation containing quercetin, luteolin, and rutin showed amended autistic activities. Additionally, compared to the initial phase of the trial, their blood levels of IL-6 and TNF-α dramatically dropped **(Table 2)** 144,164.

Research has shown that some polyphenols possess the ability to have a substantial influence on cognitive domains, including attention function and working memory. The potential neurotherapeutic actions of polyphenols are thought to be facilitated via many mechanisms, including the enhancement of cerebral blood flow and the promotion of connection within the hippocampus. Additionally, polyphenols may mitigate OS and suppress neuroinflammation, both associated with cognitive functions such as memory and frontal executive function. Sustained consumption over an extended period is required to get discernible outcomes. The advantages mentioned above may also have relevance for those in lower age groups, and there is much current study into the impact of consuming polyphenols on older persons who are at risk for dementia or AD. There is a potential correlation between the consumption of well-balanced and nourishing food and the potential improvement of age-related cognitive decline throughout the early stages of life 148,165,166.

## Toxicity of polyphenols

While most polyphenols are deemed harmless, toxic polyphenol-drug reactions, liver disease, body and weight gain, adenocarcinoma, headache, diarrhea, yellow poop, and rash have all been linked to dietary polyphenol intake or exposures. Several experimental studies have evaluated the primary adverse effects of polyphenols (**Table 3**). The research was conducted by Majid et al. to analyze how different amounts of vitamin E affected the oxidative status, semen characteristics, and trace minerals of Beetal Bucks. Thirty-six bucks of equal size and age (1 year) were randomly split into four classes. For two months, one participant received no supplementation (group 1), while the others received 200 (group 2), 400 (group 3), and 800 IU (group 4) vitamin E/animal/day. Semen samples were gathered and analyzed at the end of the experiment. The use of 800 IU/buck/day did not increase the consistency of the sperm. 174. DNA damage-induced toxicity was found in human RPE cells at a concentration of 50 µM by fisetin and luteolin 175. Very high intake of caffeic acid via oral administration may suppress the implantation of embryos and fetal weight gain 176. Administration of high quercetin doses led to decreased POLG expression and increased TFAM expression in female rats 177. Oral resveratrol administration at a dose of 0, 300, 1000, and 3000 mg/day resulted in dilatation of the renal tubules, cardiac inflammation, acute inflammation in the pelvic area, necrosis of the papillary, and severe nephropathy 178. Intake of apigenin in Swiss rats may result in oxidative stress that induces liver damage 179. In another study, genistein was administered in Sprague-Dawley rats at different doses, and adenocarcinoma occurred 180.

Another polyphenol, naringenin, has been found to decrease biodegradation 181. In mice, the LD50 of protocatechuic acid was found to be 800 mg/kg by i.p. and 3.5 g/kg by i.v. The oral LD50 of protocatechuic aldehyde in mice was 1.7 g/kg. In mice, a toxic oral dosage of 500 mg/kg causes GSH degradation of the liver and kidney but no mortality. Protocatechuic acid is a nontoxic and comparatively stable agent for oral administration due to its poor oral absorption 182. Curcumin was found to cause headache, diarrhea, yellow stool, and rash in humans at a dose of 500-12,000 mg/day 183. Consumption of polyphenols may have anti-nutritional effects. The simultaneous consumption of tea can inhibit nonheme iron absorption. Consumption of polyphenols at high doses may raise the risk of iron depletion in individuals with marginal iron status. Though it shows little toxicity, overexposure to it may be deadly or can cause death 184. To prevent liver toxicity and non-specific side effects, strategies like targeted NF-κB inhibition, direct delivery systems like nanoparticles or antibody-drug conjugates, combination therapy, dose optimization based on individual factors like disease severity and genetic variations, and biomarkers can be employed 185,186. These strategies aim to minimize off-target effects, reduce systemic exposure and side effects, enhance efficacy, and minimize side effects while maintaining therapeutic efficacy 187. Biomarkers can also predict patients likely to experience adverse effects from NF-κB inhibitors 188.

## Concluding remarks and future visions

Polyphenols, a dietary supplement with potential neuroprotective properties, encounter numerous challenges in transforming into efficacious therapies for NDs. These factors include limited absorption in the gastrointestinal tract due to their significant dimensions and attraction to dietary fiber, substantial gastrointestinal tract and liver metabolism, and the highly specific BBB. Clinical studies often produce mild or ambiguous conclusions, and focusing on a solitary molecule may not be adequate to modify an illness's advancement substantially. Furthermore, polyphenols are present in various forms and combinations in different dietary sources, which poses challenges in establishing standardized doses and comparing findings across research. Excessive use of some polyphenols might potentially interact with drugs or have negative effects on those with certain health issues. Insufficient research funding also impedes progress and the conduct of clinical studies. Continual research is being conducted to enhance the therapeutic efficacy of polyphenols, including their modification, combination with other pharmaceuticals or lifestyle interventions, and customization of therapy according to individual variables and disease subtypes.

Recently, there has been a flow in the patenting of polyphenols and their synthetic derivatives as pharmaceutical agents to treat diverse human ailments. These substances have demonstrated significant potential for both avoiding and slowing down neurodegenerative diseases. The prevention or delay of the development of NDs may be achieved by the blockade of NF-κB stimulation by polyphenols. Adequate preclinical investigations have been undertaken, demonstrating substantial improvements in neurological state. While this review examines the clinical state and includes some clinical reports, it is significant to note that these trials alone do not provide enough evidence for reaching definitive conclusions on applying these findings to human well-being. Additional clinical studies are required to validate their efficacy. Addressing the essential pharmacokinetic parameters is vital to preparing for trials adequately. It is vital to use comprehensive inquiry models that adopt a multidisciplinary strategy, including epidemiological, clinical, and molecular investigations. The effectiveness of these polyphenols has to be assessed in animal models that mimic neurological disorders and in a controlled animal model specifically designed to simulate chronic neuroinflammation. If these trials provide positive results, they might potentially demonstrate a reduction in pro-inflammatory mediators controlled by NF-κB and enhancements in cognition and memory. This implies that plant polyphenols can function as disease-modifying drugs for NDs.

## Figures and Tables

**Table 1:** *In vivo* experimental data regarding the use of polyphenols in neurological disorders

Diseases	Polyphenols	Dose Used	Study Model	Findings	Ref.
Alzheimer's disease	Resveratrol	100 µM	Adult male SD rats	Resveratrol's protective effect against Aβ-induced neurotoxicity in mice is achieved by inhibiting iNOS synthesis.	58
	LD55	-	Transgenic AD mice	Both resveratrol and LD55 showed comparable reductions in Aβ plaque load and neuroinflammation.	60
	Curcumin	150 mg/kg	APP/PS1 mice	Curcumin reduced neuroinflammation and improved neuronal function in individuals with AD.	65
		1.5 and 3 mg/kg	Male IcrTacSam: ICR mice	Epigallocatechin-3-gallate effectively inhibits cognitive dysfunction in mice by modifying secretase activity and inhibiting the ERK and NF-κB pathways.	73
	Gallic acid	10 or 30 mg/kg B.W	Mice	Gallic acid effectively inhibited neuronal cell death in an *in vivo* setting by suppressing cytokine production and reducing NF-κB acetylation levels.	72
Parkinson's disease	Hyperoside	0-20 μM	BV2 microglial cells	Hyperoside reduces the activation of microglia in neurodegenerative conditions like PD.	76
	Curcumin	40 mg/kg b.w	Aged male SD rats	Curcumin effectively reduced the protein activity of the transcription factor NF-κB, produced by LPS.	77
	Quercetin	5, 10, 20 µg/ml	SK-N-MC cell line and SD male rat	Quercetin significantly reduced manganese-induced apoptosis and inflammation in both SK-N-MC cells and SD rats, likely due to activation of the HO-1/Nrf2 and suppression of NF-κB pathways.	78
	Chlorogenic acid	25, 50, and 100 mg/kgb.w	Swiss albino male mice	Chlorogenic acid has been seen to effectively inhibit neuroinflammation in the SN region of the brain via the regulation of NF-κB expression.	79
	Baicalein	0-400 mg/kg b.w	SD rats	Baicalein, a compound with anti-neuroinflammatory properties, offers protection against brain damage caused by rotenone.	81
	Rosmarinic acid	4 mg/kg and 16 mg/kg	Male C57BL/6 mice	The use of rosmarinic acid in animal models of PD has been found to suppress NF-κB nuclear expression, potentially enhancing its anti-PD properties.	80
	Glaucocalyxin B	20-80 mg/ml	Sprague-Dawley (SD) rats	Glaucocalyxin B effectively alleviated PD symptoms by modulating the NF-κB pathways.	82
	Eriocalyxin B	0.25 μM	male C57BL/6 mice	Eriocalyxin B effectively mitigates dopamine neuron degeneration in PD models by targeting p65 phosphorylation in microglia.	83
	Calycosin	15 or 30 mg/kg/day	healthy male mice	Calycosin has been found to reduce PD symptoms in mice by regulating TLR/NF-κB and MAPK pathways.	84
	Chrysin	10 mg/kg	male C57B/6J mice	The administration of chrysin orally avoided the changes caused by 6-OHDA.	89
Cerebral Ischemia	Naringenin	10-50 mg/kg	Male Wistar rats	The administration of naringenin enhanced functional results and mitigated ischemic brain damage via inhibiting neuroinflammation mediated by NF-κB.	90
	Genistein	2.5-10mg/kg	Adult male C57/BL6J mice	Genistein's neuroprotective effect in temporary ischemia is possibly due to its ability to modulate mitochondrial apoptotic pathways and inhibit ROS-generated NF-κB activation.	91
	Catechin Hydrate	20 mg/kg b.wt	male Wistar rats	Catechin Hydrate's administration protected the brain from injury caused by MCAO, possibly due to the downregulation of NF-kB expression.	92
	Luteoloside	20-80 mg/kg	Male SD rats	The stimulation of NF-κB signaling was dramatically reduced by luteolysis.	93
Anxiety and depression	Resveratrol	40 mg/kg	C57BL/6J mice	Resveratrol, when stimulated by the Sirt1/NF-κB pathway, reduced inflammation and alleviated anxiety and depression-like symptoms.	94
		80 mg/kg	Rats	Resveratrol administration in rats with chronic unpredictable mild stress showed antidepressant effects due to inhibition of neuroinflammation, reduction of OS, apoptosis, and up-regulation of BDNF and β-catenin levels.	95
		50 mg/kg	C57BL/6J mice	The administration of resveratrol inhibited the generation of inflammatory cytokines by increasing the levels of Sirt1.	96
	Proanthocyanidin	80 mg/kg	male ICR mice	LPS upregulates iNOS and COX-2, but proanthocyanidin decreases this by inhibiting NF-κB in the hippocampus, prefrontal cortex, and amygdala.	97
	Curcumin	100 mg/kg	SD rats	Curcumin effectively reduced the expression of proinflammatory cytokines and inhibited NF-κB activation.	98
Spinal cord injury	Oleuropein	20-100 μg/kg	Mice	The use of oleuropein aglycone reduced inflammatory response in the SCI model.	99
	Resveratrol	200 mg/kg	Rat	Resveratrol significantly restores dorsal neuronal function in rat's post-spinal cord injury due to its anti-oxidative, anti-inflammatory, and anti-apoptotic properties.	100
		60 or 90 mg/kg	Rat and mouse	Resveratrol has significant efficacy in the prevention of subarachnoid hemorrhage.	101
		10 mg/kg	Mouse	Resveratrol reduces intracerebral hemorrhage-induced neurodegeneration, cerebral edema, and acute neurological deficits.	102
		400 mg/kg	SD rats	Resveratrol therapy reduced sub-lesional bone loss in spinal cord-injured mice.	103
	Eugenol	25 and 50 mg/kg	SD rats	Eugenol's neuroprotective effect against traumatic SCI may be supported by its ability to down-regulate NF-κB and MAPK signaling pathways.	104
	Curcumin	200 mg/ kg/day	Wistar albino rats	Curcumin efficiently protects spinal cord tissues from OS.	105
		100 mg/kg	SD rats	Curcumin significantly reduced NF-κB activation and inflammatory cytokine production in the injured spinal cord by inducing Nrf2 activity.	106
		100 mg/kg	SD rats	Curcumin administration after SCI significantly reduced the expression of key factors linked to the TLR-4/NF-κB inflammatory signaling pathway.	107
	Apocynin	50 mg/kg	SD rats	Apocynin administration reduced OS, mitigated neuronal apoptosis, suppressed the inflammatory response, and improved locomotor activity.	108
Autism spectrum disorder	luteolin and apigenin	20-80 mg/kg	Rat	Luteolin and apigenin could augment social deficiencies, repetitive behavior, and impairments in learning and memory.	109
	Luteolin	1 mg/kg	Mouse	Lowered the levels of NF-kB, GFAP, iNOS, and IL-1β, improved social and nonsocial behaviors	110
	7,8-dihydroxyflavone	80 mg/L	mouse	The dimension of neuronal nuclei was seen to be increased	111
	Hesperetin	10-20 mg/kg	Rat	The intervention resulted in enhancements in interpersonal skills and reductions in recurrent behaviors.	112

**Table 2:** Clinical reports regarding the use of polyphenols in neurological disorders

Number of subjects	Treatment	Doses	Duration	Findings	Ref.
24 healthy males	240 ml FR orange juice	272 mg	2-weeks	Flavonoid-rich orange juice improves subjective and objective cognition in healthy middle-aged people for 6 hours.	159
52 subjects	Decaffeinated green tea catechins	336.4 mg	12 weeks	Consuming green tea catechins daily could have positive benefits on working memory.	160
30 healthy adults	Cocoa flavanols	520 mg and 994 mg	3 days	Cocoa flavanol beverages significantly reduced self-reported mental weariness, but this effect was only observed when the beverage was consumed.	161
37 healthy older adults	Flavanone	305 mg	8-weeks	An eight-week diet high in flavanones significantly improved global cognitive performance compared to a control diet low in flavanones.	162
50 children	Luteolin + quercetin + quercetin glycoside rutin	100 mg + 70 mg + 30 mg	26 weeks	Polyphenolic therapy improved communication, focus, and collaboration while reducing aberrant behaviors.	163
24 healthy adults	Flavanone	70·5 mg	1 week	The digit Symbol Substitution Test improved significantly, although other cognitive tests did not.	167
250 patients	Palmitoylethanolamide and luteolin	10:1 by mass	60 days	Neurological status, cognitive impairment, spasticity, pain, and daily life independence all showed statistically significant increases.	168
191 females	Soy-derived isoflavones	80 mg per day	6 months	Cognitive performance is unchanged	169
78 females	Cocoa polyphenols, single dose	250 or 500 mg per day	30 days	Cognitive performance is unchanged	170
50 females	Isoflavone supplement	60 mg per day	6 weeks	Improvement in visual recall and sustained attention	171
34 adults	Soy isoflavones	116 mg per day	12 weeks	Spatial working memory improved, but auditory and episodic memory did not.	172
27 participants	EGCG	135 or 270 mg		Cognitive performance is unchanged.	173

**Table 3:** Toxicity of polyphenolic compounds based on research studies

Polyphenols	Subjects	Dose	Route of Administration	Toxic Effects	References
α-tocopherol	36 Beetal buck	0, 200, 400, 800 IU/buck/day	Oral	The use of 800 IU/buck/day did not increase the consistency of the sperm.	174
Fisetin and luteolin	Human RPE cells	50 µM	-	DNA damage-induced toxicity in human RPE cells	175
Caffeic acid	Rats	0, 0.15, 5 or 150 mg/kg/day	oral	Affected implantation of embryos and fetal weight gain	176
Quercetin	Female ICR mice	100 mg/kg	oral	The decrease in POLG expression and TFAM expression was significantly increased	177
Myricitrin	Sprague-Dawley rats	250, 500 and 1000 mg/kg/bw	oral	Increased food consumption and decreased body weight gain	189
Resveratrol	Rats	0, 300, 1000, and 3000 mg/day	oral	Dilatation of the renal tubules, cardiac inflammation, acute inflammation in the pelvic area, necrosis of the papillary, and severe nephropathy	178
Apigenin	Swiss mice	25, 50, 100 and 200 mg/kg	oral	The oxidative stress-induced liver damage.	179
Genistein	Sprague-Dawley rats	5, 100, or 500 ppm	oral	Resulted in adenocarcinoma	180
Protocatechuic aldehyde	Mice	800 mg/kg	i.p.	GSH degradation in the liver and kidneys	182
		3.5 g/kg	i.v.		
		1.7 g/kg	oral		
Curcumin	Seven humans	500-12,000 mg	oral	Experienced headache, diarrhea, yellow stool, and rash	183
	Human	0.45 to 3.6 g/day		Diarrhea and nausea and an increase in lactate dehydrogenase and serum alkaline phosphatase contents	

POLG= polymerase gamma, TFAM= transcription factor A
